# Analysis of Array-CGH Data Using the R and Bioconductor Software Suite

**DOI:** 10.1155/2009/201325

**Published:** 2009-08-19

**Authors:** Winfried A. Hofmann, Anja Weigmann, Marcel Tauscher, Britta Skawran, Tim Focken, Reena Buurman, Luzie U. Wingen, Brigitte Schlegelberger, Doris Steinemann

**Affiliations:** Hannover Medical School, Institute of Cell and Molecular Pathology, Carl-Neuberg-Str. 1, 30625 Hannover, Germany

## Abstract

*Background*. Array-based comparative genomic hybridization (array-CGH) is an emerging high-resolution and high-throughput molecular genetic technique that allows genome-wide screening for chromosome alterations. DNA copy number alterations (CNAs) are a hallmark of somatic mutations in tumor genomes and congenital abnormalities that lead to diseases such as mental retardation. However, accurate identification of amplified or deleted regions requires a sequence of different computational analysis steps of the microarray data. *Results*. We have developed a user-friendly and versatile tool for the normalization, visualization, breakpoint detection, and comparative analysis of array-CGH data which allows the accurate and sensitive detection of CNAs. *Conclusion*. The implemented option for the determination of minimal altered regions (MARs) from a series of tumor samples is a step forward in the identification of new tumor suppressor genes or oncogenes.

## 1. Introduction

The goal of array-CGH experiments is to detect and map genetic changes involving copy number aberrations of chromosomal segments at a high resolution along the genome. The computational analysis of the experimental data involves the preprocessing of raw microarray data, aligning data with genome location, identification of altered chromosomal segments, assignment of a copy number state to each segment, and highlighting of regions repetitively changed across several experiments. There are numerous methods and software packages freely available to analyze array CGH data, many of which are listed in the review by Lockwood et al. [1]. The acgh software can be downloaded from the Hannover Medical School website http://www.mhh.de/acghtool.html. One widely used software suite, collecting many new algorithms and methods for the analysis of genomic data, including array CGH data, is the open source Bioconductor project [2, 3], which is embedded in the open source statistical programming environment R [4]. The methods for array-CGH analysis available from this platform are described as follows. 

The MANOR package (Microarray Normalization of array-CGH data) provides tools to normalize array-CGH data. This includes the possibility to add flags for spot filtering, to calculate scores for array quality, and particularly to apply a new normalization algorithm to correct for local spatial bias and continuous spatial gradients [5]. Two methods were available for smoothing array-CGH data, namely, quantsmooth, using a quantile smoothing method [6] and waveslim, which is part of the R project, using smoothing algorithm denoising by wavelets [7]. Segmentation or breakpoint detection methods detect copy number states of chromosomal sections and locate positions of transition between sections of different copy number states. The DNAcopy package uses the Circular Binary Segmentation algorithm for this task [8, 9].

 CGHcall uses the segmentation results of DNAcopy and determines for each segment the most likely copy number [10]. The GLAD package implements a Gaussian-based approach that models a piecewise constant function based on the Adaptive Weights Smoothing procedure (Polzehl) [11]. In addition, a state (gain, amplification, single or double loss) is assigned to each segment. Picard et al. suggest a Gaussian model and estimate the number of segments by an adaptive penalty function which has been integrated into the tiling Array package [12, 13]. A method based on hierarchical clustering along chromosomes is used in the CLAC package [14]. Hidden Markov models, where copy numbers are hidden states, are used in the aCGH package [15], and BioHMM extends the algorithm of Fridlyand et al. by taking the distance between clones and other variables into account [16]. 

cghMCR helps to locate minimum common regions (MCRs) altered across different samples. The breakpoint detection method of the DNAcopy package defines altered regions when a threshold value is provided, by selecting a subset of altered and identifying overlapping groups of segments [17]. The snapCGH package integrates methods from other packages for normalization and breakpoint detection and supplements these with additional algorithms and functions. It supports different microarray platforms, currently Agilent, BlueFuse, and Nimblegen, provides normalization methods from the limma package, and allows averaging over triplicate spots. Moreover, several breakpoint detection methods can be applied from the packages aCGH, DNAcopy, GLAD, and tilingArray. In addition, a new breakpoint detection method, BioHMM, is implemented [16]. The package provides advanced graphical output, for example, plotting log ratios for each clone against its position on the genome. Of particular use is the possibility to have all results of different breakpoint detection algorithms in one graph. The performance of several commonly used segmentation algorithms is compared in Lai et al. who test several algorithms for smoothing and breakpoint detection on simulated and real data [18, 19]. For the breakpoint detection methods, the algorithm developed by Picard et al. and DNAcopy produced the best results concerning the tradeoff between true and false positive detection rates of clones assigned to an aberrant state when tested on the simulated data. However, the algorithm developed by Picard et al., as well as DNAcopy, GLAD, and a genetic search algorithm, GA, performed very well in detecting known aberrations in real tumor data [20]. Willenbrock and Fridlyand tested breakpoint detection methods and the subsequent assignment to gain and loss status of three algorithms, namely, aCGH, DNAcopy, and GLAD [19]. They developed a new method to create simulated data of similar complexity to real array CGH data. Moreover, they developed new segment-merging algorithm assigning to each segment a copy number status. In their test, DNAcopy performed best with the highest sensitivity and the lowest false discovery rate for breakpoint detection. Results were similar with the new segment-merging algorithm or the segment-merging algorithm from the GLAD package.

Our goal was to develop a user-friendly routine for the automatic analysis of the BAC/PAC array described in Zielinski et al. [21], which bundles the different analysis steps and can be routinely run by the researchers in the lab. We used bioconductor and the statistical programming language R, since they provide a wide choice of widely used methods and algorithms described above for preprocessing and analyzing array CGH data. Moreover, the flexibility of R allows methods to be combined, adapted, or to have new modules added to them. Our analysis routine reads in the raw array CGH data, preprocesses and normalizes them using the marray package [22], creates diagnostic plots to check array quality, performs breakpoint detection using either the GLAD program directly [11] or different segmentation algorithms of the snapCGH package, and provides additional options to summarize the analysis results and to locate regions aberrant across several arrays.

## 2. Materials and Methods

We installed R, version 2.8.1 [4] on a Pentium IV PC with 2.4 GHz and 1 GB RAM as a hardware minimum configuration running both Microsoft Windows 2000 SP4/XP SP2 and Linux 2.6.2x. On Windows, the binary distribution was used, whereas on Linux, the source was compiled according to the instructions in the INSTALL file. Bioconductor, version 2.3, the GLAD, and the snapCGH packages were installed according to the instructions [3]. 

### 2.1. Array CGH

DNA chips containing 6934 individual BAC/PAC (DKFZ, Heidelberg, Germany) clones spotted in triplicate were used. They allow a genomewide resolution of 1 Mb and an even higher resolution of up to 100 kb for chromosome regions recurrently involved in human tumors as well as for regions containing known tumor suppressor genes and oncogenes. Isolation and spotting of DNA probes were performed as described previously [21]. Probe processing and image analysis were carried out as published earlier [23]. Data normalization and computational analysis is described during the text. The information on chromosome lengths and positions of the cytogenetic bands were obtained from the UCSC genome browser using the human genome assembly of March 2006 [24].

### 2.2. Fluorescence In Situ Hybridization (FISH)

FISH was performed as described [25]. For the detection of the Cyclin D1 gene and the LSI IGH/CCND1 Dual Color, Dual Fusion Translocation Probe (Abbott Molecular Inc., Des Plaines, IL, USA) was used with the CCND1 probe labeled with Spectrum Orange and the IGH probe with Spectrum Green.

## 3. Results and Discussion

To demonstrate the functionality of the routine we used array-CGH data gained from 34 patients with hepatic tumors as described earlier [23]. DNA arrays used contained 6934 BAC/PAC clones spotted in triplicate. The genomewide average resolution was 1 Mb, with some regions known to contain tumor suppressor genes or oncogenes or regions known to be recurrently involved in human tumors, having a resolution of up to 100 kb.

As a first step, raw array data were preprocessed, and the quality of the almost 20 000 spots and of the whole arrays was assessed. On startup, the digital read-outs obtained from the imaging analysis software (GenePix 4000 A; Axon Instruments, Union City, CA, USA), a file containing a sorted list of the BAC clones including their position on the chromosome and genes contained in them, and a file with the array layout (location of the BAC clones on the array) are read in. During preprocessing, the routine automatically chooses spots of good quality which will be used for the further analysis. The quality criteria for retaining a spot were as follows: the signal intensity of the spot must be at least three times the background intensity, and the absolute ratio of mean and median pixel intensity of a spot must be at least 0.85. As clones were spotted in triplicate on the array, the signal intensities were calculated as mean intensities of the three spots. A further quality criterion is that at least two of the three spots have to fulfill these quality criteria, and the standard deviation between triplicates must be lower than 0.2. The R routine produces statistics on array quality and further details (settings of GenePix software, percentage of high quality clones on each array, mean intensity of signals per array).

**Figure 1:**
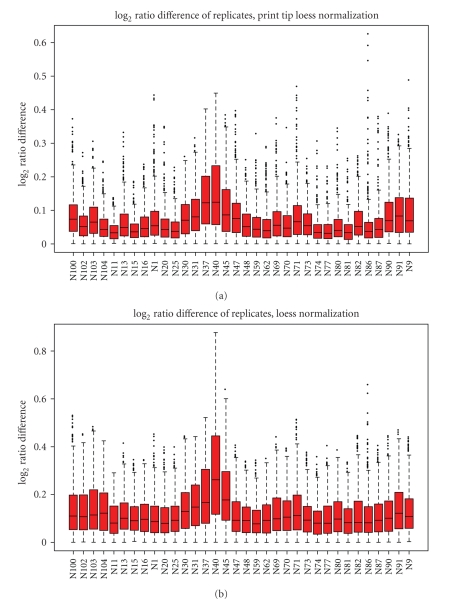
Boxplots showing experimental variability between replicates for each array. Log_2_ ratio differences of clones spotted at two different locations on the array are smaller for all arrays after print tip loess normalization (a) than after loess normalization (b). The individual arrays are listed along the *X*-axis, and the *Y*-axis shows the difference between the log_2_ ratios of clones spotted at two different positions on the array.

Following the preprocessing, normalization is carried out using the marray package. The user can interactively choose between methods available in marray. The marray diagnostic plots to check for array quality are automatically generated by the routine. An additional diagnostic plot for the specific array type was developed. On these arrays, 459 clones are spotted at two different positions on the array. The routine determines the log_2_ ratio differences of the replicates and produces a graph with boxplots of these differences for each array (Figure 1). In the case of a perfect array, these replicates would produce identical values. Any deviations will demonstrate a less than optimal array quality. However, normalization can reduce this problem, the effect of different normalization methods can be tested, and the most suitable one can be chosen.

The effect of normalization is demonstrated in Figure 1 where the log_2_ ratio differences of the replicates are smaller after print tip loess normalization than after loess normalization. For this batch of arrays, print tip loess was chosen as the default normalization method.

In order to determine regions of chromosomal gain or loss, breakpoint detection is carried out next. We have tested three breakpoint detection algorithms available in R, namely, aCGH, DNAcopy, and GLAD. DNAcopy performed best in the comparative studies as described above, whereas GLAD is particularly strong in detecting very small aberrations of 2-3 clones, which comes at the cost of a higher false positive rate. The false positive rate can be reduced by considering only small aberrations detected at a particular position across several samples. We have integrated the breakpoint detection algorithm and graphical presentations of the GLAD package in the routine. The assignment of a state to a segment (gain, amplification, single or double loss) can be done by either using the algorithm of GLAD or by choosing a threshold value.

**Figure 2:**
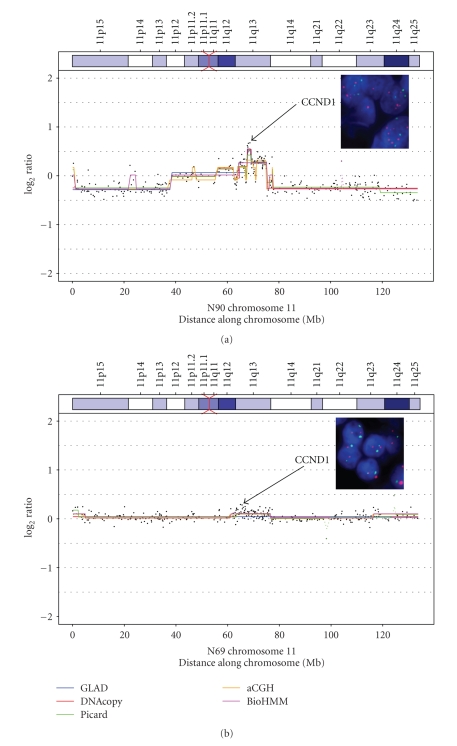
Copy number states of chromosome 11. Copy number states of chromosome 11 as assigned by five different breakpoint detection algorithms (GLAD, DNAcopy, Picard, aCGH, BioHMM) are shown for two hepatocellular tumor cases, N90 and N69. FISH analyses on formalin-fixed paraffin-embedded tissue sections are shown in the inset box; orange spots: CCND1 probe, green spots: IGH probe.

As another option, the snapCGH package was integrated into the routine, as this package offers several breakpoint detection methods (e.g., GLAD, DNAcopy, Picard, aCGH, BioHMM), and the results of several methods can be summarized in a single graph. The representation of the chromosomes and cytobands (obtained from the GLAD package) was integrated in the graphs. In Figure 2, a comparison of the five breakpoint detection methods is shown for chromosome 11. For case N90, different copy number states along chromosome 11 are indicated with the highest log_2_ ratio within 11q13 containing the *CCND*1 gene. The most sensitive methods detecting this amplification were GLAD and DNAcopy, which assign an additional segment with a log_2_ ratio state ≥0.5 for six BAC clones. To check whether this tiny segment reflects a true amplification or an artifact caused by outliers, we performed FISH on paraffin sections. Figure 2 shows the result of this hybridization performed on two cases, N90 and N69. A clear amplification of *CCND*1, as indicated by six or more red signals, can be seen in N90, whereas N69 shows a normal signal constellation of two red and two green signals, verifying gains in N90 and the normal state in N69, as predicted by the software.

**Figure 3:**
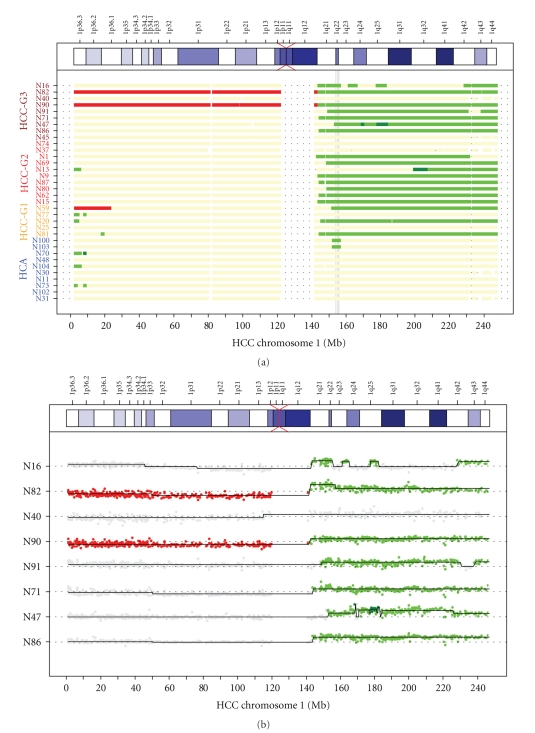
Overview of copy number states of chromosome 1 of several hepatocellular samples. Breakpoint detection was performed with GLAD; thresholds for assigning copy number states were 0.2 and −0.2 for gain and loss, respectively, 0.6 for amplification, and −0.6 for homozygous loss. In (a) only states are shown, in (b) states and original log_2_ ratios are plotted for a selection of samples. In (a) a minimally altered region (MAR) present in 19 of the 34 arrays is indicated by a gray vertical bar. Red indicates loss, green gain, and dark green amplification. Base pair position along chromosome 1 is plotted on the *X*-axis (Mbp scale at the bottom, cytogenetic banding at the top); samples are shown along the *Y*-axis.

The comparison of the chromosomal alignment of several aCGH experiments in a single graph was the ultimate goal of the computational analysis as a tool to find minimally altered regions across arrays. The following analysis steps were thus included in the routine: the detection of MARs by cghMCR package, with the possibility to choose the number of arrays in which the aberration should at least be present, and the generation of list of BAC clones including chromosomal position, log_2_ ratio, segment log_2_ ratio, segment copy number state, outlier status, genes contained, and breakpoint position (data not shown). An example of an overview showing copy number states for chromosome 1 across several experiments is presented in Figure 3(a). 

**Figure 4:**
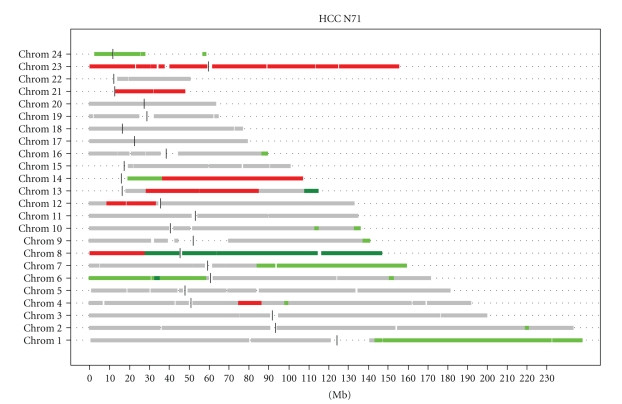
Copy number states summarized for all chromosomes of sample N71. Breakpoint detection was performed as in Figure 3. Thresholds for assigning copy number states were 0.2 and −0.2 for gain and loss, respectively, 0.6 for amplification, and −0.6 for homozygous loss. Red indicates loss, green gain, and dark green amplification. Vertical bars show the location of the centromeres. Base pair position along the chromosomes is plotted on the *X*-axis; chromosomes are stacked vertically in numerical order (1 to 24, whereby 23 is chromosome X, and 24 is chromosome Y).

The position of a minimally altered region within cytoband 1q22, present in 19 samples, is indicated. In Figure 3(b), the log_2_ ratios are shown for a subset of arrays. This allows the visualization of different levels of segment log_2_ ratios within an assigned copy number status. An additional option in our routine is to display an overview of the copy number states for all chromosomes of each single experiment in a single graph as shown in Figure 4. 

Information on location of breakpoints and log_2_ ratio values of segments is summarized for each array, as exemplified in Table 1 in Supplementary Material available online at doi:10.1155/2009/201325 (see also Table 1). A summary of those clones contained in a MAR can also be generated (data not shown). 

## 4. Conclusions

We have developed a semiautomatic routine that reads in raw array CGH data, preprocesses and normalizes them using the marray package, creates diagnostic plots to check array quality, performs breakpoint detection using either the GLAD package directly or different segmentation algorithms included in the snapCGH package, locates regions aberrant across several arrays, and summarizes the results of the analysis.

## Supplementary Material

Information on size and values of segments after breakpoint detection is summarized for each array, as exemplified for sample N16 shown in Supplementary Table 1.

## Figures and Tables

**Table 1:** Information on segments after breakpoint detection shown for chromosome 11 of sample N16. The columns give information about smoothing value, gain or loss (where gain is 1, loss is −1, 2 is more than 3-fold gain, −2 is homozygous loss, and normal state is 0), kb_start and kb_end which give the exact Mb position of breakpoint start and end, and banding_start and banding_end of the corresponding chromosomal bands.

Chromosome	Smoothing	Gain/loss	kb_start	kb_end	Banding_start	Banding_end
11	−0.11	0	223	44963	p15	p11.2
11	0.386	1	46300	47384	p11.2	p11.2
11	−0.032	0	47956	72088	p11.2	q13
11	−0.353	−1	72273	72273	q13	q13
11	−0.755	−2	72476	72651	q13	q13
11	−0.098	0	72857	97216	q13	q22
11	0.133	0	98400	98400	q22	q22
11	−0.132	0	99685	134070	q22	q25
