# Supplementary material for: Analysis of Array-CGH Data Using the R and Bioconductor Software Suite
Source: Comp Funct Genomics. 2009 Aug 19;2009:201325. doi: 10.1155/2009/201325 (PMC2728899; doi:10.1155/2009/201325)
Supplement: Supplementary file 1 — Information on size and values of segments after breakpoint detection is summarized for each array, as exemplified for sample N16 shown in Supplementary Table 1. [file 201325.f1.pdf]

Supplementary Table1

Information on segments after breakpoint detection shown for sample N16. The columns give information about chromosomes 1 to 24 (whereby 23 is X and 24 is Y), smoothing value, gain or loss (where gain is 1, loss is -1 and normal state is 0), kb_start and kb_end give the exact Mb position of breakpoint start and end, and banding_start and banding_end the corresponding chromosomal bands..

chromosome	smoothing	gain/loss	kb_start	kb_end	banding_start	banding_end
1	0.144	0	1032	45691	p36.3	p34.1
1	-0.036	0	45806	75909	p34.1	p31
1	-0.19	0	76691	142715	p31	q21
1	0.417	1	143566	155323	q21	q23
1	-0.047	0	156057	160977	q23	q23
1	0.392	1	161301	165055	q23	q24
1	-0.055	0	165441	177454	q24	q25
1	0.386	1	177629	182125	q25	q25
1	-0.048	0	182574	227684	q25	q42
1	0.348	1	229057	247097	q42	q44
2	0.141	0	315	202677	p25	q33
2	0.02	0	203318	242568	q33	q37
3	-0.081	0	243	104130	p26	q12
3	0.034	0	105264	150639	q13.1	q25
3	-0.103	0	151152	165377	q25	q26.1
3	0.035	0	166995	199069	q26.1	q29
4	0.044	0	131	2829	p16	p16
4	0.248	1	3454	16083	p16	p15.3
4	0.086	0	17067	55786	p15.3	q12
4	-0.111	0	55922	121157	q12	q27
4	-0.214	-1	121230	190979	q27	q35
5	0.072	0	1341	50001	p15.3	q11.1
5	-0.253	-1	50115	180570	q11.1	q35
6	0.015	0	149	29408	p25	p22
6	0.228	1	31733	33542	p21.3	p21.3
6	0.727	2	33629	33945	p21.3	p21.3
6	0.467	1	34284	34985	p21.3	p21.3
6	0.128	0	35487	35487	p21.3	p21.3
6	0.621	2	35661	44322	p21.3	p21.1
6	0.215	1	44425	62656	p21.1	q11
6	-0.011	0	63844	127455	q12	q22
6	0.146	0	128541	170732	q22	q27
7	0.015	0	223	158577	p22	q36
8	-0.311	-1	568	6685	p23	p23
8	-0.701	-2	7243	7949	p23	p23

8	-0.278	-1	8104	34371	p23	p12
8	0.014	0	34572	121349	p12	q24.1
8	-0.14	0	122799	142055	q24.1	q24.3
8	0.013	0	142184	146085	q24.3	q24.3
9	-0.266	-1	50	29566	p24	p21
9	0.148	0	33066	38062	p13	p13
9	-0.476	-1	43329	43329	p11	p11
9	0.036	0	70404	140065	q13	q34
10	0.042	0	312	42746	p15	q11.2
10	-0.25	-1	43945	54007	q11.2	q21
10	-0.095	0	54114	135346	q21	q26
11	-0.11	0	223	44963	p15	p11.2
11	0.386	1	46300	47384	p11.2	p11.2
11	-0.032	0	47956	72088	p11.2	q13
11	-0.353	-1	72273	72273	q13	q13
11	-0.755	-2	72476	72651	q13	q13
11	-0.098	0	72857	97216	q13	q22
11	0.133	0	98400	98400	q22	q22
11	-0.132	0	99685	134070	q22	q25
12	0.11	0	153	51930	p13	q13
12	0.27	1	52009	54924	q13	q13
12	-0.222	-1	54928	54928	q13	q13
12	0.37	1	55152	55787	q13	q13
12	0.124	0	55820	56044	q13	q13
12	0.437	1	56148	56202	q13	q13
12	0.084	0	56203	110418	q13	q24.1
12	-0.046	0	110782	131946	q24.1	q24.3
13	0.038	0	18674	51548	q12	q14
13	-0.028	0	52270	108864	q21	q33
13	0.052	0	109014	113828	q33	q34
13	0.334	1	113863	113863	q34	q34
13	0.056	0	114017	114017	q34	q34
14	-0.174	0	19651	39454	q11.2	q21
14	-0.107	0	40463	106227	q21	q32
15	-0.093	0	19881	99969	q11. 2	q26
16	0.064	0	102	88552	p13.3	q24
17	0.119	0	392	20171	p13	p11.2
17	-0.032	0	20325	78374	p11.2	q25
18	0.448	1	213	5005	p11.3	p11.3
18	-0.217	-1	5352	6843	p11.3	p11.3
18	0.851	2	6843	14066	p11.3	p11.2
18	0.162	0	14066	17460	p11.2	q11.2
18	0.699	2	17993	22836	q11.2	q11.2
18	-0.278	-1	23747	75940	q12	q23
19	0.009	0	211	23644	p13.3	p12
19	0.311	1	32995	63687	q12	q13.4
20	0.343	1	230	25023	p13	p11.2
20	0.003	0	26113	29580	p11.1	q11.2
20	0.319	1	29779	62393	q11.2	q13.3

21	-0.115	0	13545	46846	q11.2	q22
22	-0.034	0	14497	49243	q11.1	q13
22	0.287	1	49426	49453	q13	q13
23	-0.226	-1	536	2706	p22.3	p22.3
23	0.159	0	3031	92222	p22.3	q21
23	0.35	1	93081	154664	q21	q28
24	-0.751	-2	2931	57461	p11.3	q12
